# Transcriptome Analysis of Embryogenic and Non-Embryogenic Callus of *Picea Mongolica*

**DOI:** 10.3390/cimb45070332

**Published:** 2023-06-21

**Authors:** Yaping Wang, Hao Wang, Wenquan Bao, Mingming Sui, Yu´e Bai

**Affiliations:** College of Forestry, Inner Mongolia Agricultural University, Huhhot 010019, China; yapingwang113@gmail.com (Y.W.); 18832987252@163.com (H.W.); bwq@imau.edu.cn (W.B.); 15048565065@emails.imau.edu.cn (M.S.)

**Keywords:** somatic embryogenesis, callus, transcriptomics, morphology, histocytology, physiology, *Picea mongolica*

## Abstract

*Picea mongolica* is a rare tree species in China, which is of great significance in combating desertification and improving the harsh ecological environment. Due to the low rate of natural regeneration, high mortality, and susceptibility to pests and cold springs, *Picea mongolica* has gradually become extinct. At present, somatic embryogenesis (SE) is the most effective method of micro-proliferation in conifers, but the induction rate of embryogenic callus (EC) is low, and EC is difficult to differentiate from non-embryonic callus (NEC). Therefore, the EC and NEC of *Picea mongolica* were compared from the morphology, histological, physiological, and transcriptional levels, respectively. Morphological observation showed that the EC was white and transparent filamentous, while the NEC was compact and brownish-brown lumpy. Histological analyses showed that the NEC cells were large and loosely arranged; the nuclei attached to the edge of the cells were small; the cytoplasm was low; and the cell gap was large and irregular. In the EC, small cells, closely arranged cells, and a large nucleus and nucleolus were observed. Physiological studies showed significant differences in ROS-scavenging enzymes between the EC and NEC. Transcriptome profiling revealed that 13,267 differentially expressed genes (DEGs) were identified, 3682 were up-regulated, and 9585 were down-regulated. In total, 63 GO terms had significant enrichment, 32 DEGs in plant hormone signal transduction pathway were identified, and 502 different transcription factors (TFs) were characterized into 38 TF families. Meanwhile, we identified significant gene expression trends associated with somatic embryo development in plant hormones (*AUX*/*IAA*, *YUCCA*, *LEA*, etc.), stress (*GST*, *HSP*, *GLP*, etc.), phenylpropanoid metabolism (*4CL*, *HCT*, *PAL*, etc.), and transcription factors (*AP2*/*ERF*, MYB, *WOX*, etc.). In addition, nine genes were chosen for RT-qPCR, and the results were consistent with RNA-Seq data. This study revealed the changes in morphology, histology, physiology, and gene expression in the differentiation of NEC into EC and laid the foundation for finding the key genes to promote EC formation.

## 1. Introduction

*Picea mongolica* W. D. Xu, only distributed in the southeast edge of Otindag Sandy Land in Inner Mongolia, China, is a rare tree species with capabilities of cold tolerance, drought tolerance, and sand-burying tolerance [1]. This wood is mainly used for papermaking, furniture, and musical instrument making, and turpentine can be extracted from its bark. At the same time, as a natural protective barrier, the *P. mongolica* forest plays a very important role in promoting local agricultural production and the ecological environment in North China [2]. However, due to the low rate of natural regeneration, high mortality, and susceptibility to pests and cold springs, *P. mongolica* is gradually becoming extinct. In addition, due to the huge genome and high heterozygosity of *P. mongolica*, seed breeding will inevitably lead to the separation of excellent traits [3]. Therefore, the establishment of somatic embryo regeneration and breeding systems in *P. mongolica* can accelerate the cultivation of seedlings and also lay the foundation for the genetic improvement of modern molecular breeding technology in *P. mongolica*.

Plant somatic embryogenesis (SE) is a process in which a single cell or tissue directly induces a callus in vitro without fertilization and eventually differentiates into a whole plant under suitable conditions [4]. Due to its relative genetic stability, repeatability, and efficiency, SE has become an important biotechnology tool and has been widely used in germplasm storage [5], high-quality seedling production [6], artificial seeds [7], and molecular and cell engineering breeding [8,9]. In an experiment on the SE induction of conifers, Durzan and Chalupa first observed the embryoid structure of *Pinus banksiana* in an in vitro culture [10]. Subsequently, among Pinaceae plants, including *Pinus strobus*, *loblolly pine*, and *Pinus pinaster*, somatic embryos were successfully induced from immature zygotic embryos [11,12,13]. In particular, Picea trees, including *Picea morrisonicola* [14], *Picea koraiensis* [15], *Picea likiangensis* [16], *Picea abies* [17], and *Picea rubens* [18], have reported successful SE regeneration. In our previous study, we established a complete SE regeneration system for *P. mongolica* [19] but found that the induction cycle of its embryogenic callus (EC) is long and the induction efficiency is low. Therefore, a further understanding of the molecular mechanism and key genes involved in SE is necessary to shorten the period of somatic embryo regeneration.

Previous studies have revealed that the EC induction process is controlled by complex genetic factors, including plant hormone-related genes, stress-related genes, and transcription factors [20]. The essential stages of SE are characterized by the induction of the expression of numerous plant hormone-related genes, including *PIN* [21], *ARF* [22], *YUCCA* [23], and *AUX*/*IAA* genes [24]. *Germin-like proteins* (*GLPs*) were specifically expressed at the initial stage of somatic embryo induction and differentiation in Arabidopsis [25], wheat [26], and cotton [27], and studies in conifers showed that *LmGER1* was specifically expressed at the root cap of young embryos [28]. Heat Shock Protein 90 (Hsp90), which is involved in plant developmental processes [29,30,31], showed low expression throughout the early stages of the embryo but was strongly expressed during embryo maturation in Arabidopsis [32]. Many *GSTs* were found in barley, cotton, and soybean to participate in their embryonic development processes [33,34,35]. In addition, several transcription factors are critical in regulating SE, such as *WUSCHEL* (*WUS*) [36], *LEAFY COTYLEDON* (*LEC*) [37], *AGAMOUS-Like* (*AGL15*) [38], and *BABY BOOM* (*BBM*) [39]. However, the drivers of EC induction in *P. mongolica* have not yet been reported.

In this study, the morphology, cytology, and physiology of the non-embryonic callus (NEC) and EC of *P. mongolica* were observed, and the gene expression profiles were analyzed by RNA-seq technology. The major candidate genes were identified and discussed in the NEC vs. the EC. This will enhance our knowledge of the SE of *P. mongolica* and provide theoretical guidance towards solving the problem of EC induction in SE.

## 2. Materials and Methods

### 2.1. Plant Materials

*P. mongolica* seeds, collected from Keshiketeng Banner, Chifeng City, Inner Mongolia Autonomous Region, China, were stored in a refrigerator at 4 °C.

### 2.2. Callus Induction and Stress Treatment

The mature seeds were soaked in clean water for 24 h. After surface sterilization with 2% NaClO for 6–8 min and sterile water, the mature embryos isolated from the seeds were cultured in induction medium (Table S1). After 28 days, the brown part of the radicle was excised and cultured again in the proliferation medium and supplemented with 5% polyethylene glycol (PEG) 6000 and 20 mM NaCl induction medium, respectively, and the proliferation rate of the EC was counted. After 25 days, the EC and NEC were separated and cultured in the proliferation medium, respectively (Table S1). The EC that grew well in the proliferation medium were selected and put into the differentiation medium for embryoid induction (Table S1). All the cultures were maintained in the dark at 25 ± 1 °C.

### 2.3. Histological Observation

After culturing on the proliferation medium for 25 days, fresh EC and NEC were selected and fixed in FAA. After washing with 50% alcohol three times, the calli were stained with hematoxylin-eosin at 37 °C for three days. After dehydration with a graded ethanol series (70–100%) and transparent treatment with a xylene series (50–100%), the calli were then embedded in paraffin and sliced using a slicer, with each slice being 10 μm thick. Finally, the sections were sealed with glue, and the morphological differences were observed under the light microscope.

### 2.4. Physiological Determinations

To determine the activity of the antioxidant enzymes, the EC and NEC were taken at 25 days (0.1 g per sample, 3 replicates) and immediately stored at −80 °C until they were used for the physiological assays. In particular, the activity of superoxide dismutase (SOD), peroxidase (POD), catalase (CAT), ascorbate peroxidase (APX), and polyphenol oxidase (PPO) was determined according to standard methods (Solarbio, Beijing, China).

### 2.5. RNA-seq

The total RNA was extracted from the EC and NEC using TRIzol^®^ Reagent (Invitrogen, CA, USA). The RNA quality was determined using 1% agarose gels and a 2100 Bioanalyzer (Agilent, CA, USA). RNA-Seq libraries were constructed at Beijing Novogene Technology Co., Ltd. (Beijing, China) using the NEBNext^®^ UltraTM RNA Library Prep Kit (NEB, MA, USA) according to the manufacturer’s recommendations. Finally, the library was performed on the HiSeq2500 platform for high-throughput sequencing.

### 2.6. Identification of Differentially Expressed Genes (DEGs) and Enrichment Analysis

Through CASAVA base recognition, the high-throughput sequencer transforms the image data of the sequenced fragment into sequence data (reads). The file is in the format of fastq, which mainly contains the sequence information of the sequenced fragment and its corresponding sequence quality information. The reads with the adapter, the reads with *n*, and the low-quality reads were removed. HISAT2 v2.0.5 (https://ccb.jhu.edu/software/hisat2/index.shtml, accessed on 28 May 2023) was used to compare the paired terminal clean reads with the Norway spruce genome (PlantGenIE.org: Home, accessed on 8 July 2020) to obtain mapped reads. FPKM was used to express the gene expression, and DESeq2 software was used for differential expression analysis. The multiple comparison detection *p*-values described by Benjamin and Yekutieli [40] were used to check the error detection rate FDR [41,42,43]. The DEGs with *p* < 0.01 and |log2FC| ≧ 1.5 were considered to be significant. Goseq R and KOBAS software were used to enrich and analyze the DEGs, such as GO and KEGG. TBtools software [44] was used to draw a heatmap according to the gene expression level.

### 2.7. Quantitative Real-Time PCR (qRT-PCR)

qRT-PCR was carried out to verify the reliability of the RNA-seq data, and the EF1α from *P. mongolica* was used as the reference gene. Specific primers for 11 DEGs related to EC development for qRT-PCR are listed in Table S2. The qRT-PCR was run on the iCycler iQ system (Bio-Rad, CA, USA) using SYBR qPCR Master Mix (Vazyme, Nanjing, China) according to the manufacturer’s instructions. Three biological and technical repetitions were used for each sample. The relative expression of the DEGs from the qRT-PCR was calculated with the 2^−ΔΔCT^ method. GraphPad. Prism 9.0 (Graphpad, CA, USA) was used to draw histograms according to the gene expression level.

## 3. Results

### 3.1. Morphological and Histological Analysis of EC and NEC

**Figure 1:**
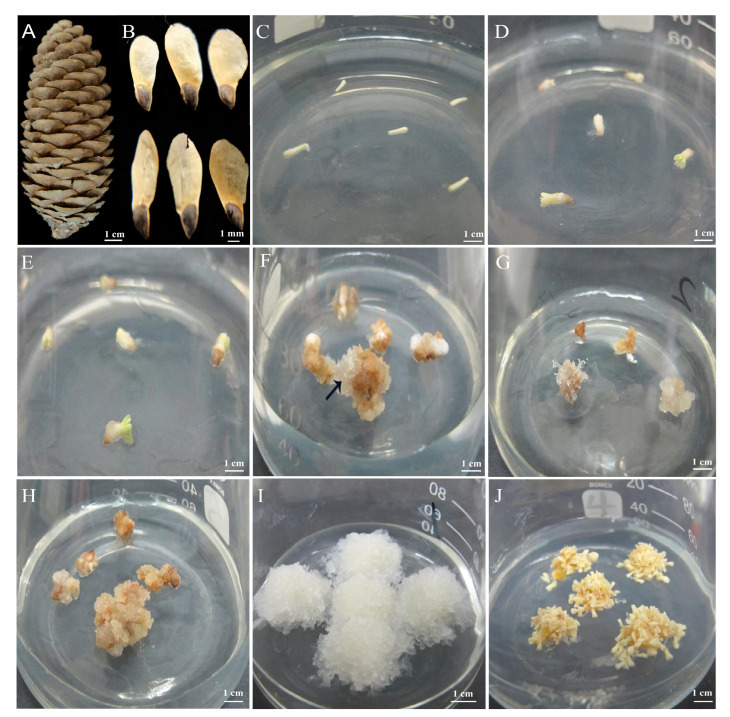
Callus formation from mature zygotic embryos of *Picea mongolica*. (**A**) Cones. (**B**) Maturing seeds. (**C**–**F**) Mature somatic embryos were inoculated on induction medium for 0, 6, 9, and 28 days. (**G**) Proliferation of white filiform callus after subculturing. (**H**,**I**) NEC and EC grown on 25 days of proliferation medium. (**J**) The embryo is produced by differentiation of the embryogenic callus.

To obtain the EC and NEC of *P. mongolica*, mature embryos were stripped from the sterilized seeds and placed on the induction medium (Figure 1A–C). After induction for 6 days, the whole embryo begins to expand; the radicle and cotyledon turn brown and green, respectively (Figure 1D). A callus began to appear at the hypocotyl on the 8th day (Figure 1E). With an increase in the induction time, the callus became larger, and until the 28th day, white filamentous calli grew on the edge or surface of the massive calli (Figure 1F). After subculturing, the white, transparent, filamentous callus began to grow rapidly (Figure 1G). Finally, the NEC and EC were separated and inoculated in the proliferation medium to make them proliferate and grow stably (Figure 1H,I). In addition, the differentiation of EC can produce a large number of embryoids (Figure 1J).

**Figure 2:**
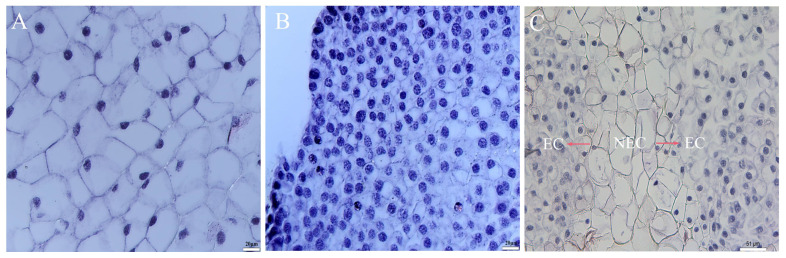
Microstructure observation of the callus of *Picea mongolica*. (**A**) NEC. (**B**) EC. (**C**) EC grows on the surface of NEC.

Histological analysis showed that the NEC cells were large cells with a loose cell arrangement without rules, a small nucleus attached to the cell edge, and a large gap junction (Figure 2A), whereas the EC cells had a large nucleus with a close cell arrangement (Figure 2B). The inner layer of the cell was mostly ordinary parenchyma cells, and the EC appeared outside the cell mass, which can develop into somatic embryos. In addition, the EC was produced on the surface or edge of the NEC (Figure 2C). Therefore, we speculate that the SE of *P. mongolica* is of external origin.

### 3.2. The Identification, GO Classification, and KEGG Enrichment Analysis of DEGs

A total of 30,681 DEGs were expressed in both the EC and NEC, and 7012 and 2495 DEGs were uniquely expressed in the NEC and EC, respectively (Figure S1). Additionally, 3682 genes were up-regulated and 9585 genes were down-regulated in the EC compared with the NEC (fold change > 1.5; *p*-value < 0.05; Figure S2). GO enrichment analysis showed that 34, 10, and 19 GO terms were significantly enriched in biological processes, cell components, and molecular functional classification. Among them, 3914 DEGs were classified into biological processes, mainly enriched in chemical processes, oxidative stress, and metal ion transport. In total, 994 DEGs were classified into cellular components, mainly in the cell periphery, cell wall, and external encapsulating structure; a total of 5529 DEGs were classified into molecular functions, mainly in transcriptional regulator activity, DNA-binding transcription factor activity, and antioxidant activity (Figure S3).

A total of seven pathways were significantly enriched, including photosynthesis, phenylpropanoid biosynthesis, plant hormone signal transduction, phenylpropanoid biosynthesis, the plant hormone signal transduction pathway, photosynthesis-antenna proteins, plant-pathogen interaction, starch and sucrose metabolism, and glutathione metabolism (*p*-value < 0.05). Strikingly, 32 DEGs are annotated into the plant hormone signal transduction pathway, which is the most annotated pathway of differential genes (Figure S4), indicating that deciphering the discrepancies between the EC and NEC was critical to understanding what enables the NEC to obtain pluripotency and further differentiate into EC.

### 3.3. Identification and Expression Pattern Analysis of Important DEGs in the Plant Hormone Pathway

**Figure 3:**
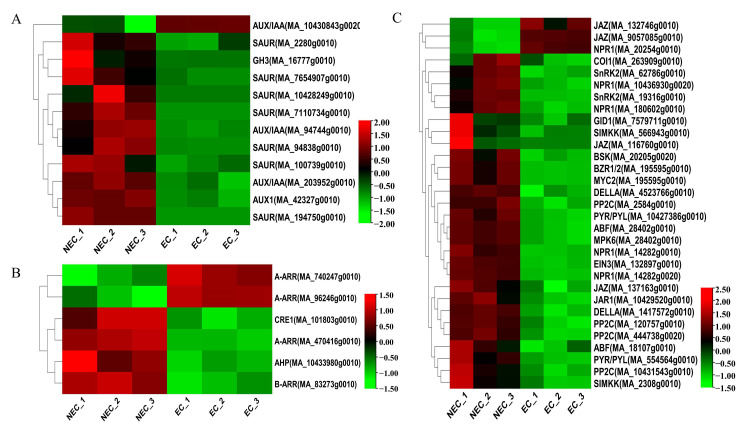
Identification and expression profile of the DEGs annotated to the hormone synthesis and signal pathways. (**A**) Auxin signal transduction. (**B**) Cytokinin signal transduction. (**C**) Pathways of other hormones, such as gibberellin, abscisic acid, ethylene, brassinolide, salicylic acid, and jasmonic acid.

Plant hormone-related genes play a key role in SE [45]. In our study, numerous genes involved in auxin (12 DEGs), cytokinin (6 DEGs), gibberellin (3 DEGs), abscisic acid (10 DEGs), ethylene (4 DEGs), brassinolide (2 DEGs), salicylic acid (5 DEGs), and jasmonic acid (7 DEGs) biosynthesis and signal transduction pathways were differentially expressed in the EC vs. the NEC. For example, the levels of expression of AUX/IAA (MA_10430843g0020) increased from the NEC to EC. Nevertheless, SAUR (MA_100739g0010, MA_194750g0010, MA_7110734g0010, MA_2280g0010, MA_94838g0010, MA_10428249g0010, MA_7654907g0010), AUX1 (MA_42327g0010), and AUX/IAA (MA_203952g0010, MA_94744g0010) were significantly up-regulated (Figure 3A). Cytokinin signal transduction-related DEGs, such as A-ARR (MA_740247g0010, MA_96246g0010), were up-regulated, while CRE1 (MA_101803g0010), AHP (MA_10433980g0010), A-ARR (MA_470416g0010), and B-ARR (MA_83273g0010) were down-regulated (Figure 3B). For jasmonic acid biosynthesis, JAZ (MA_132746g0010, MA_9057085g0010) was up-regulated, while the transcripts, including JAZ (MA_116760g0010, MA_137163g0010), JAR1 (MA_10429520g0010), COI1 (MA_263909g0010), and MYC2 (MA_195595g0010), were down-regulated in the EC vs. NEC. In salicylic acid signal transduction, except for NPR1 (MA_20254g0010), the other four NPR1 genes (MA_10436930g0020, MA_14282g0010, MA_14282g0020, and MA_180602g0010) had low expression in the EC. Gibberellin, abscisic acid, ethylene, and brassinolide signal-related genes decreased from the NEC to EC (Figure 3C). Overall, in the EC vs. the NEC, most of the DEGs had 43 genes that were down-regulated, especially AUX/IAA, SAUR, PP2C, and NPR1, which were 2, 7, 4, and 4, respectively, while 6 genes were up-regulated, of which AUX/IAA, JAZ, NPR1, and A-ARR were 1, 2, 2, and 2, respectively. Therefore, plant hormone-related genes may be the key regulator genes for the EC formation of *P. mongolica*.

### 3.4. Identification and Expression Pattern Analysis of Important DEGs in the Phenylpropanoid Metabolism Pathway

Our KEGG enrichment found significant differences in the DEGs between samples in the phenylpropane metabolism pathway (Figure S4). Furthermore, the 8, 1, 1, 5, and 6 DEGs were involved in regulating 4-coumarate CoA ligase (4CL), Shikimate O-hydroxy cinnamoyl transferase (HCT), Scopoletin glucosetransfer (SGT), Cinnamoyl CoA reductase (CCR), and Beta glucose, respectively. However, the 14, 1, 6, 7, and 36 DEGs were down-regulated. Interestingly, the DGEs involved in regulating the levels of phenylalanine amine lyase (PAL), Caffeoyl shikimate esterase (CSE), and Caffeoyl CoA O-methyltransferase (CCoAOMT) were all down-regulated, including 3, 4, and 4, respectively (Figure S5).

### 3.5. Identification and Expression Profile Analysis of Putative Decisive DEGs Related to Stress Responses

**Figure 4:**
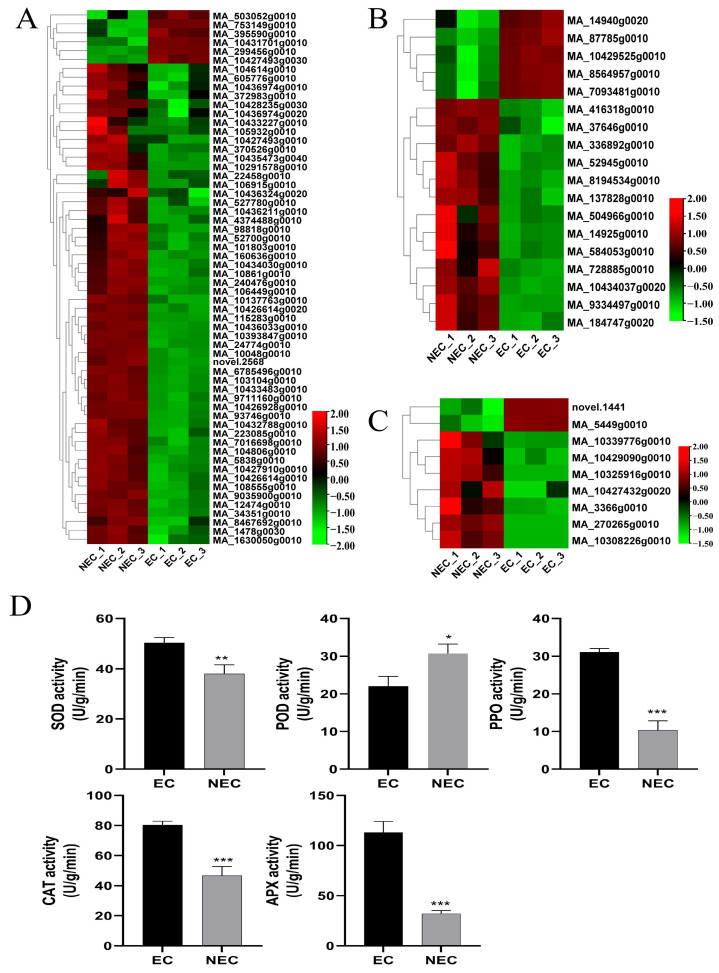
Differential analyses of the stress response of the EC and NEC of *Picea mongolica*. (**A**) The heatmap of DEGs related to HSP. (**B**) The heatmap of DEGs related to GST. (**C**) The heatmap of DEGs related to GLP. (**D**) Antioxidant enzyme activity (*, *p* < 0.05, **, *p* < 0.01, ***, *p* < 0.001).

Stress-related genes have been shown to play a vital role during SE [46]. A total of 87 DEGs were identified and analyzed for gene expression, including GST, HSP, and GLP in the EC vs. the NEC (Figure 4A–C). Furthermore, the levels of expression of five GST genes were up-regulated, and MA_7093481g0010 showed the highest up-regulated expression, while 13 GST genes were down-regulated, and MA_10434037g0020 showed the highest down-regulated expression. Moreover, nine GLP genes were identified, of which two were up-regulated and seven were down-regulated. In addition, a large number of HSP genes were identified in the EC vs. the NEC; in total, 5 were up-regulated and 55 were down-regulated. MA_299456g0010 and MA_240476g0010 showed the highest levels of up-regulation and down-regulation, respectively. Meanwhile, the activities of SOD, CAT, PPO, and APX in the EC of *P. mongolica* were significantly higher than those in the NEC, while the POD activity was higher in the NEC than in the EC (Figure 4D). Among them, the APX enzyme activity of the EC exceeded 100, which was more than three times that of the NEC. These results suggested that the NEC is more susceptible to the physiological factors of browning. Interestingly, we analyzed 100 DEGs enriched in the ROS pathway and found that most of them were related to the antioxidant enzymes POD, SOD, CAT, GPX, and APX (Figure S6). In addition, we conducted drought and salt stress treatments on the callus in Figure 1G and found that appropriate stress can effectively promote EC formation and growth (Figure S7). The above results showed that a large number of stress genes were involved in the expression of EC differentiation in *P. mongolica*.

### 3.6. Identification and Expression Profile Analysis of Expression of Decisive Transcription Factors (TFs) Associated with SE

**Figure 5:**
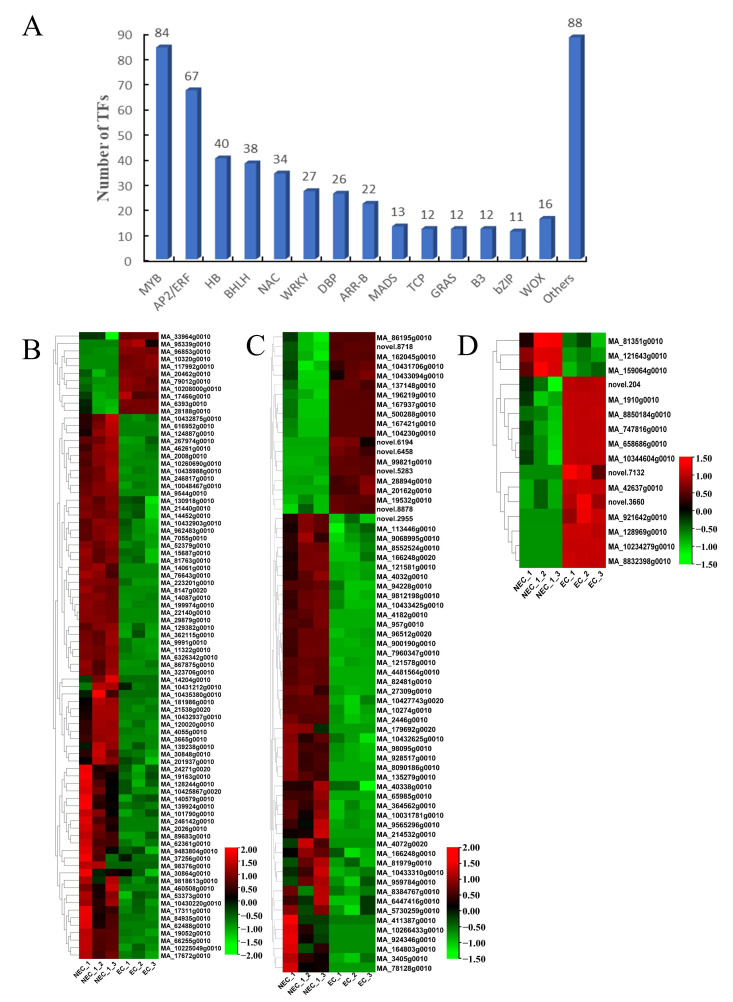
Identification and expression profile of the TFs annotated to SE in EC vs. NEC. (**A**) Identification and categorization of TF families in EC vs. NEC. (**B**) The heatmap of DEGs related to MYB. (**C**) The heatmap of DEGs related to AP2/ERF. (**D**) The heatmap of DEGs related to WOX.

In total, 502 TFs belonging to 38 different TF families were identified in the EC vs. the NEC, mainly in the MYB, AP2/ERF, HB, BHLH, and NAC families (Figure 5A). Furthermore, we analyzed the differential expression levels of the MYB, AP2/ERF, and WOX families in the EC vs. the NEC (Figure 5B–D). The levels of expression of most of the MYB, AP2/ERF, and WOX genes were up-regulated in the EC vs. the NEC. In particular, MYB3 (MA_8147g0020), AP2 (MA_8090186g0010), BBM2 (MA_86195g0010), and WOX4 (MA_121643g0010) had the highest down-regulation expression in the EC vs. the NEC, while MYB305 (MA_10320g0010), ERF071 (MA_196219g0010), and WOX8 (MA_10234279g0010) had the highest up-regulation expression in the EC vs. the NEC. In addition, our results showed 29 down-regulated genes, of which ABI3, ERF, IAA, BRI1, CYP, LEA, YUCCA, ARF, and PIN were 6, 2, 3, 4, 2, 3, 3, 3, and 3, respectively, while 30 genes were up-regulated, of which ABI3, ERF, IAA, BRI1, LEA, ARF, LEC, and PIN were 2, 2, 2, 1, 11, 2, 3, and 7, respectively (Figure S8).

### 3.7. Validation of the Expression Level of Key Candidate DEGs by qRT-PCR

**Figure 6:**
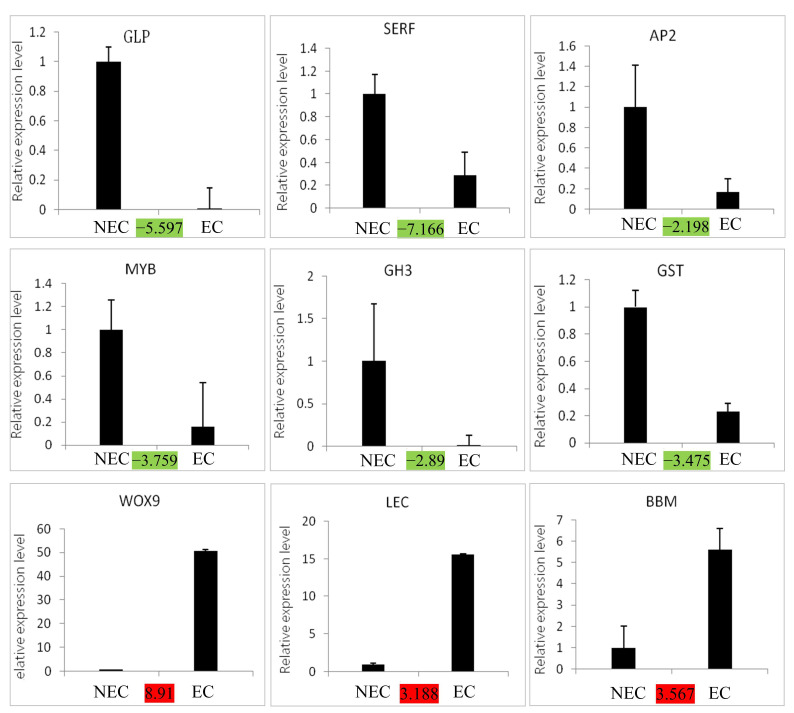
Validation of the expression of nine important candidate DEGs by qRT-PCR. Red and green symbols indicate up-regulated and down-regulated expression of transcriptome sequencing results, respectively.

In order to verify the reliability of the transcriptome data, nine key DEGs involved in SE were analyzed by qRT-PCR (Figure 6). The results showed that all nine DEGs were basically consistent with the transcriptome data, and the correlation between the transcript fold ratios and the qRT-PCR analysis was 0.82, indicating the reliability of the transcriptome data in *P. mongolica*.

## 4. Discussion

SE is currently one of the most widely used commercial methods for conifer reproduction, with four stages: EC induction, EC proliferation, SE maturation, and SE germination. EC induction is a critical step for adventitious shoot formation [47]. Many investigations have revealed that certain genes and pathways regulate plant EC induction, with hormone levels being important for EC establishment [20]. In our study, we observed and analyzed the morphological, histological, and physiological differences between the EC and NEC to investigate the mechanisms responsible for the long cycle and low induction efficiency of EC production in *P. mongolica*. Meanwhile, using RNA-seq, we compared their transcriptional patterns and identified 13,267 DEGs.

To reveal the regulatory mechanism of EC formation, 13,267 DEGs, with 3682 up-regulated and 9585 down-regulated (Figures S1 and S2) genes, were further annotated to plant hormone pathways, particularly the auxin, cytokinin, and ABA pathways, by GO and KEGG analysis (Figures S3 and S4), which is consistent with previous research [48,49], indicating the requirement of plant hormones in SE in *P. mongolica*. Furthermore, there is no genomic data for *P. mongolica*, which severely hinders research into its molecular biology. According to our findings, a total of 796.8 M reads were constructed, which will provide basic data support for *P. mongolica* molecular function research.

Auxin plays a vital role in regulating cell division, differentiation, and the establishment of embryonic polarity [49,50]. *YUC1*, *YUC4*, *YUC10*, and *YUC11*, which are members of the *YUCCAs* family that encode for important enzymes in the Trp-dependent auxin production pathway, are expressed in discrete groups of cells during embryogenesis [23].

In addition, many previous studies have shown that *GH3* [51], *ARF* [22], *CYP* [52], *AUX*/*IAA* [24], and *SAUR* [53] play a key regulatory role during SE. In our study, most of the expression levels of *YUCCA*, *GH3*, *ARF*, *IAA*, and *SAUR*, the key genes in Trp synthesis, were drastically down-regulated in the EC vs. the NEC. Only *ARF* (MA_10432580g0010, MA_2421g0010) and *AUX*/*IAA* (MA_10430843g0020, MA_9357208g0010, MA_18664g0010) showed EC specificity with high FPKM (Figure 4A and Figure S4), suggesting that the level of IAA during the NEC was higher than the EC; a low IAA level might promote the NEC to differentiate into the EC. *PIN-FORMED1* (*PIN1*), *PIN4*, and *PIN7* proteins are major factors in the maintenance of auxin gradients in the embryo [54,55]. *PIN1* (MA_100472g0010, MA_10430006g0010) and *PIN7* (MA_138314g0010) were significantly up-regulated in this study, whereas the seven identified auxin input proteins *PIN* (MA_57211g0010, novel.7303, novel.477, MA_674082g0010, novel.5169, MA_50550g0010, MA_6089g0010) were significantly down-regulated (Figure S8).

The three cytokinin-encoding receptors *CRE1*, *AHK2*, and *AHK3* were the first to be discovered in Arabidopsis and are crucial calli-generating factors [56]. On the *CRE1* receptor, MA_101803g0010 was identified and was dramatically down-regulated (Figure 3B). The downstream target of *AHP* is *ARR*, which may be classified into type A and type B based on the product’s amino acid sequence and expression pattern [57]. By controlling cell cycle-related genes in Arabidopsis, *ARR1* can further encourage callus division and growth [58]. In this study, two up-regulated expressions of type A-ARR, one down-regulated expression, and one down-regulated expression of type *B-ARR* were implicated in the cytokinin signal transduction pathway. The findings above demonstrate that ARR is a critical regulator of *P. mongolica*’s EC differentiation. Therefore, exogenous cytokinin concentrations can be controlled to hasten EC production during the callus induction of *P. mongolica*.

Genes involved in the biosynthesis and signal transduction pathways of various hormones, except auxin and cytokinin, as well as gibberellin, ethylene, brassinolide, salicylic acid, jasmonic acid, and abscisic acid were also expressed differently in the NEC and EC (Figure 3C). EC formation and SE germination are impacted by the GA3 content [59]. We discovered that the genes *GID1* and *DELLA*, which are involved in signal transduction and gibberellin production, were up-regulated in the NEC and down-regulated in the EC. SE is promoted by increased ethylene content and the activation of its signal pathway [60]. *SIMKK*, *MPK6*, and *EIN3*, DEGs associated with ethylene signal pathways, were down-regulated in the EC compared to the NEC. The expression of the ethylene-related synthesis genes *CTH*, *cysK*, *GOT1*, *EIN2*, and *EIN3* was, likewise, reduced in the EC in comparison to the NEC in upland cotton [61]. In the EC vs. the NEC, the DEGs of *BSK* and *BZR1*/*2*, which are implicated in BR signal pathways, were down-regulated. The SAR pathway’s main regulatory factor, *NPR1* (*nonexpressor of pathogenesis-related genes 1*), is crucial for plant growth and development [62,63]. Our findings showed that five *NPR1* genes were expressed differently in the EC than in the NEC. *JAR1*, *COI1*, *MYC2*, and 2 *JAZ* were down-regulated in the EC compared to the NEC, but genes linked to JA signal pathways, such as 2 *JAZ*, were up-regulated. Abscisic acid is associated with SE initiation and expression, and the transition from NEC to EC is followed by a fast increase in endogenous ABA levels [64]. Our results showed that 10 ABA signal pathway-related genes were specifically accumulated in the NEC.

One of the main mechanisms for plant stress adaptation is the phenylpropanoid pathway [65]. This pathway predominates a number of metabolic pathways involved in pathogen defense and abiotic stress, including the formation of flavonoids and lignin [66]. The three phenol propane monomers that make up the majority of lignin are crucial regulators of plant development, cellular mechanical support, and stress response [67]. A crucial enzyme in the production of lignin is phenylalanine amine lyase (PAL), also known as 4-coumarate CoA ligase (4CL) [68]. Additionally, three essential enzymes in the plant shikimate pathway—cinnamoyl CoA reductase (CCR), caffeoyl shikimate ester (CSE), and caffeoyl CoA O-methyltransferase (CCoAOMT)—are involved in the manufacture of significant phenolic acids and are recognized as potent antioxidants [69]. In maize, phenylpropanoid metabolic pathways have several DAPS that are highly abundant in the NEC, according to proteomic analysis of the EC and NEC [70], whereas, in avocado, the phenylpropanoid metabolism pathways accumulate more in the EC than in the NEC [71]. According to our research, the majority of these genes are down-regulated in the EC (Figure S5), suggesting that the NEC needs more lignin.

Stress factors play an important role in somatic embryogenesis. Many studies have proved that stress treatment, including ABA treatment [72], starvation treatment [73], osmotic stress [74], and high-temperature treatment [75], can promote SE and development. In our study, osmotic stress with 20 mM NaCl and 5% PEG6000 boosted the rate of EC proliferation (Figure S7). We measured the enzyme activities of the EC and NEC and discovered that the EC had much greater SOD, PPO, CAT, and APX enzyme activity than the NEC, but the NEC had significantly higher POD enzyme activity than the EC (Figure 4D). Additionally, several *SOD*, *POD*, *APX*, *CAT*, and *GPX* genes had distinct levels of expression in the EC compared to the NEC, with the majority of *CAT* and *SOD* genes being highly expressed in the EC and the majority of *POD* genes being highly expressed in the NEC (Figure S6). Previous studies on *Gossypium hirsutum* L. [76], *Agapanthus praecox* [49], *Araucaria angustifolia* [77], *Musa* spp. [78], and *Medicago arborea* L. [79] also demonstrated the critical function that antioxidant enzymes play in the development of somatic embryos. The crucial enzyme *glutathione-S-transferase* (*GST*), which can also contribute to the metabolism of plant hormones, protects cells by scavenging various stress-induced reactive oxygen species (ROS) [80,81]. The GST gene is essential for giving somatic cells the potential to develop into embryos and can shield plant cells from severe auxin damage [82]. In total, 18 *GST* genes, of which 5 were up-regulated and 13 were down-regulated, were found to be significantly expressed in the process of cell dedifferentiation by our investigation (Figure 4B). By contributing to the defense of ROS clearance, aiding the somatic embryo in removing too many hazardous substances, boosting cell dedifferentiation, and obtaining embryogenic capacity, *GST* is triggered by hormone stress. By controlling the cell walls, *germination-like protein* (*GLP*) can influence and participate in developing coniferous somatic cells [28]. The expression of the nine *GLP* genes discovered in this study varied substantially (Figure 4C). Our research, which found 60 heat shock protein genes, is intriguing because it shows that a variety of stressors influence callus development during callus formation and that stress-related genes are essential for callus development (Figure 4A). Three *HSP* genes were found and cloned by Donget et al. in white spruce somatic embryos, and the transcription of two of these genes was mainly concentrated in the EC. However, by the seedling stage, this gene’s expression was essentially undetectable [83].

Previous research has revealed that transcription factor initiation can govern the transition from NEC to EC or SE induction. *LAFL*, *BBM*, *AGL15*, *WIND1*, and *WOX5* are among them [20]. Our work thoroughly examined the transcription factor data of *P. mongolica* and discovered 502 differentially expressed TFs, the bulk of which belonged to the *MYB* and *AP2*/*ERF* TF families (Figure 5). *MYB*, for example, is implicated in numerous plant stress responses and promotes SE [84,85]. Furthermore, the ectopic expression of *MYB118* and *MYB115* stimulated Arabidopsis root callus formation and increased the expression of the SE maker gene *LEC1* [72]. Our findings show that 19, 11, 2, 2, 2, 1, 11, 2, 3, and 7 *AP2*/*ERF*, *MYB*, *ABI3*, *ERF*, *IAA*, *BRI1*, *LEA*, *ARF*, *LEC*, and *PIN* genes are significantly up-regulated (Figure 6 and Figure S4). Interestingly, only three of the sixteen *WOX* genes were discovered to be up-regulated in the EC, whereas the other thirteen genes were strongly expressed, implying that the *WOX* gene is required for SE in *P. mongolica*. During somatic embryogenesis, the *WUS* gene in Arabidopsis promotes the change of auxin-dependent vegetative tissue into embryonic tissue [36,86]. *WOX2* is employed as an SE marker gene in pine trees [87]. *WOX9* expression is highest during the SE of *Picea abies* and *Medicago truncatula* [88,89]. Furthermore, research has shown that ABA induces the expression of the *LEA* gene and that the exogenous injection of ABA causes a considerable up-regulation of *LEA* expression [90]. Yang et al. [91,92] discovered multiple *LEA* genes in carrot and Arabidopsis and discovered that after hormone treatment, the expression of LEA genes in embryogenic tissues increased, which can be employed as molecular indicators of SE. Two up-regulated *LEA* genes (MA_63890g0010, MA_111913g0010) were found to be strongly expressed in the EC but extremely low in the NEC (Figure S8). Thus, the LEA genes were expressed uniquely in the EC and might be employed as marker genes for *P. mongolica* EC. Furthermore, research has revealed that the *LEC* gene can boost the accumulation of stored substances and speed up the transition to embryonic cells and that the *LEC1* gene is a crucial regulatory component in embryonic development [93]. Two *LEC1* genes were found to be overexpressed in our study. We hypothesize that the *LEC1* gene promotes the accumulation of significant amounts of nutrients, such as starch, providing energy for embryonic callus formation and that it may play a positive regulatory role in embryonic callus formation.

In summary, embryogenic ability requires a large number of nutrients to provide energy, and multiple transcription factors have been identified for differential expression in EC formation. These results indicate that the formation of a regulatory network of transcription factors controls the formation of embryogenic calluses in *P. mongolica*.

## 5. Conclusions

**Figure 7:**
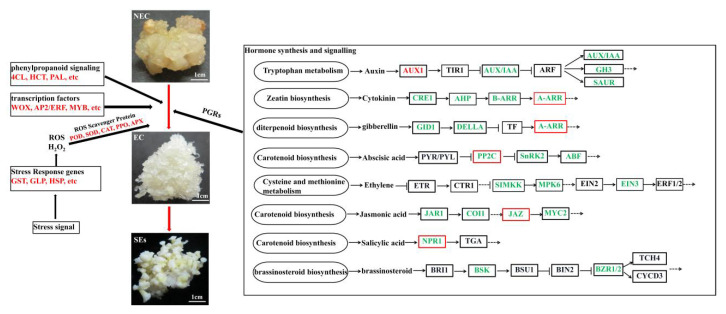
A proposed molecular regulation diagram of TFs, hormones, phenylpropanoid, and stresses in *Picea mongolica* somatic embryogenesis. Red words or lines indicate up-regulated gene expression, and green words indicate down-regulated gene expression.

We used RNA-Seq to perform a comparative transcriptome analysis of the EC and NEC derived from *P. mongolica*. This study identified some DEGs in the EC vs. the NEC and categorized them according to various biological processes that play a key role in the regulation of SE, such as hormone signaling, stress signaling, phenylpropanoid signaling, and transcription factors (Figure 7). The results of the qRT-PCR showed that the *LEC*, *WOX9*, and *BBM* genes and the *GST*, *GLP*, *SERF*, *GH3*, *AP2*, and *MYB* genes were significantly up-regulated and down-regulated in the EC vs. the NEC, respectively. However, we need to further verify the biological functions and molecular regulatory networks of these key genes through genetic transformation. In summary, our analysis of the differences in the gene profiles between the EC and NEC increased our understanding of the underlying SE in *P. mongolica* and other coniferous species.

## Data Availability

All the sequence data were deposited into the NCBI sequence read archive (SRA) under the BioProject study PRJNA974895 (Samples: SAMN35301251-SAMN35301256).

## Supplementary Materials

The following supporting information can be downloaded at https://www.mdpi.com/article/10.3390/cimb45070332/s1, Figure S1. Venn diagram of DEGs. Figure S2. Volcano plot of DEGs. Figure S3. GO enrichment analysis of DEGs. Figure S4. Scatter plot of KEGG enrichment analysis. Figure S5. Heatmap of the DEGs related to phenylpropanoid metabolism pathway in EC vs NEC. Figure S6. Heatmap of the DEGs related to the antioxidant enzymes in EC vs NEC. Figure S7. The proliferation rate of EC callus growing in induction medium for 5 to 30 days. Figure S8. Heatmap of the DEGs related to TFs annotated to SE in EC vs NEC. Table S1. Medium composition table. Table S2. qRT-PCR primer information.
