# Supplementary material for: Transcriptome Analysis of Embryogenic and Non-Embryogenic Callus of Picea Mongolica
Source: Curr Issues Mol Biol. 2023 Jun 21;45(7):5232–47. doi: 10.3390/cimb45070332 (PMC10378709; doi:10.3390/cimb45070332)
Supplement: Supplementary file 1 [file cimb-45-00332-s001.zip › cimb-2448808-supplementary/Figures S1-S8.pdf]

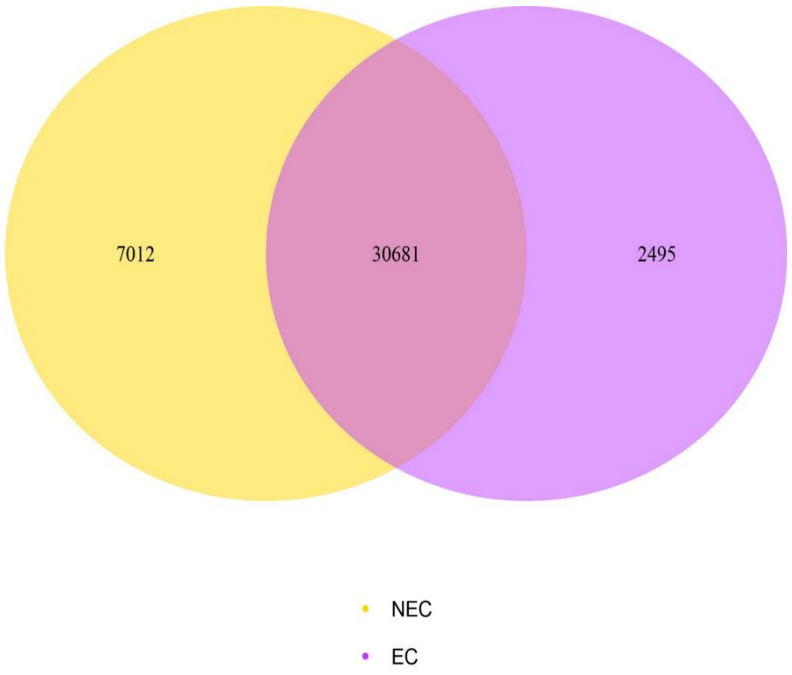

Figure S1. Venn diagram of DEGs.

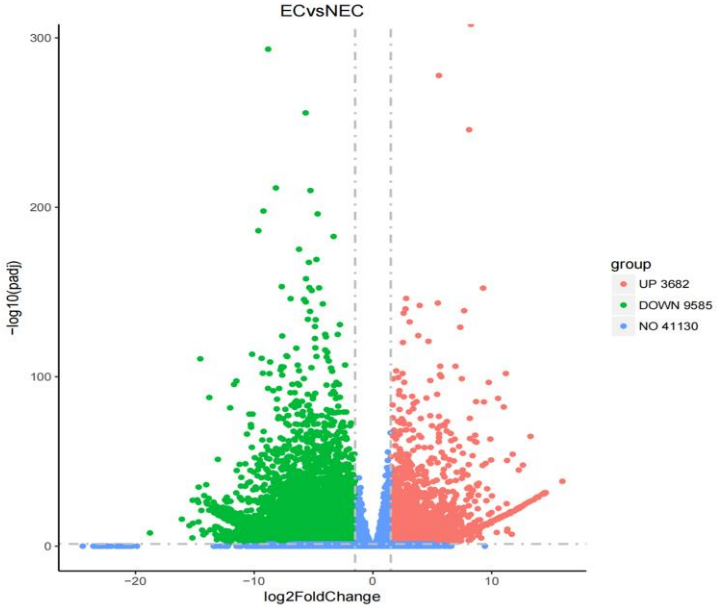

Figure S2. Volcano plot of DEGs.

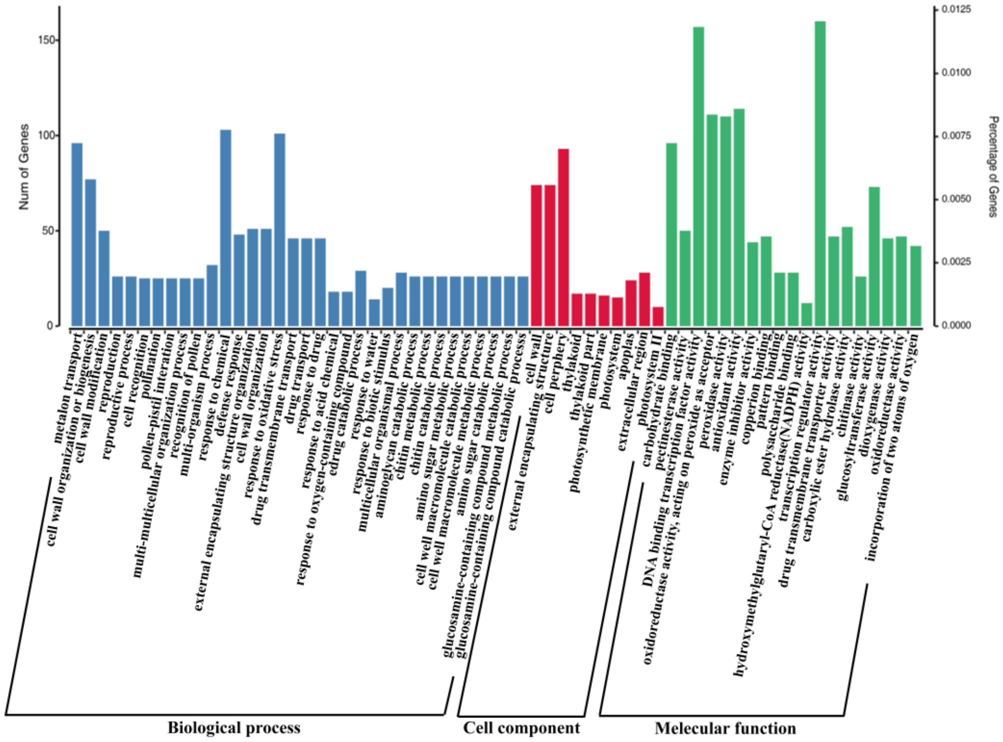

Figure S3. GO enrichment analysis of DEGs.

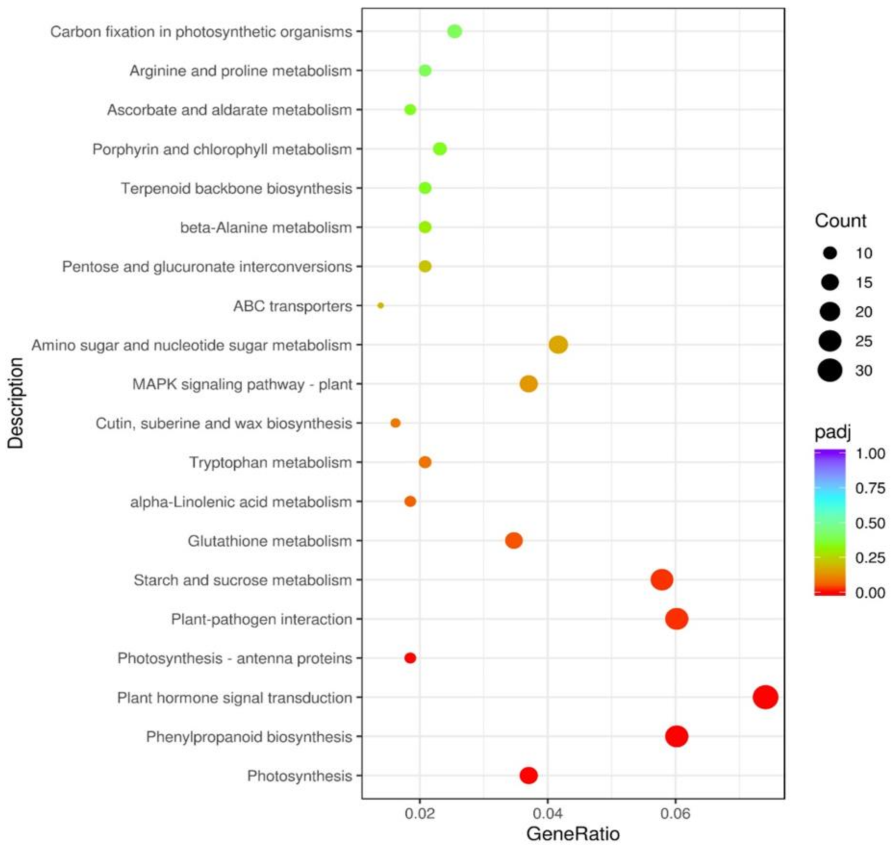

Figure S4. Scatter plot of KEGG enrichment analysis.

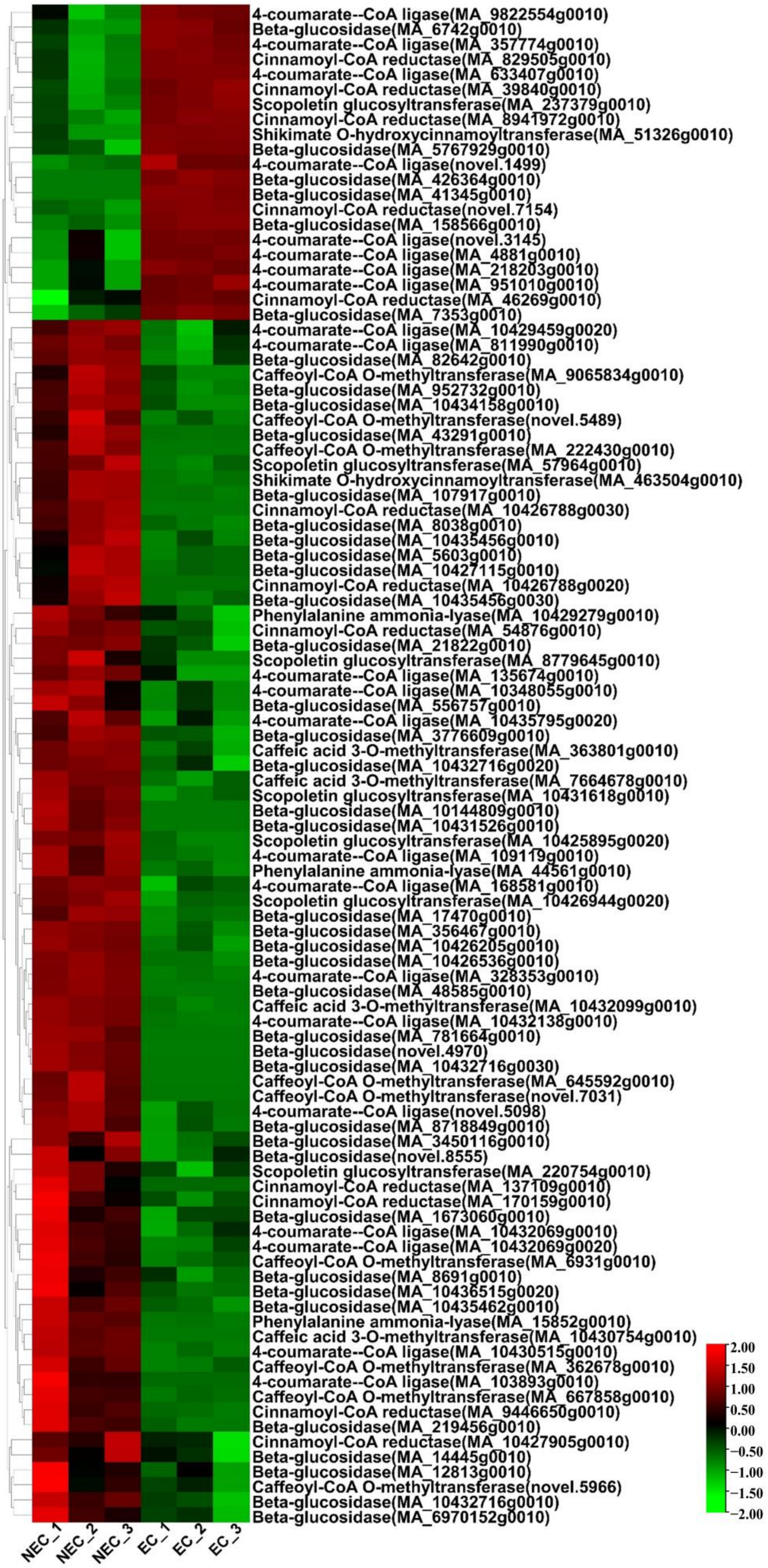

Figure S5. Heatmap of the DEGs related to phenylpropanoid metabolism pathway in EC vs NEC.

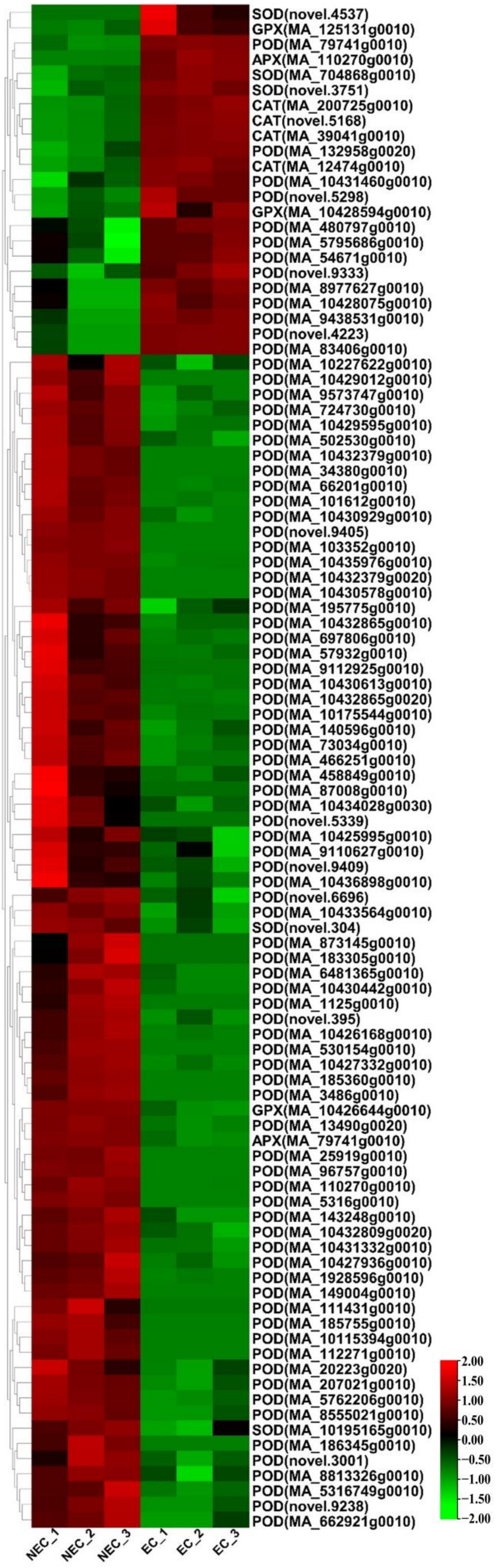

Figure S6. Heatmap of the DEGs related to the antioxidant enzymes in EC vs NEC.

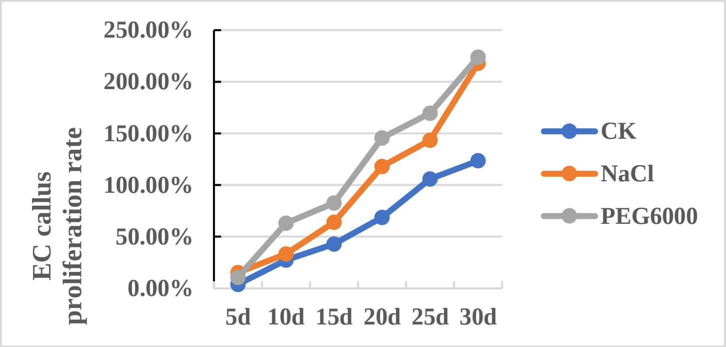

Figure S7. The proliferation rate of EC callus growing in induction medium for 5 to 30 days.

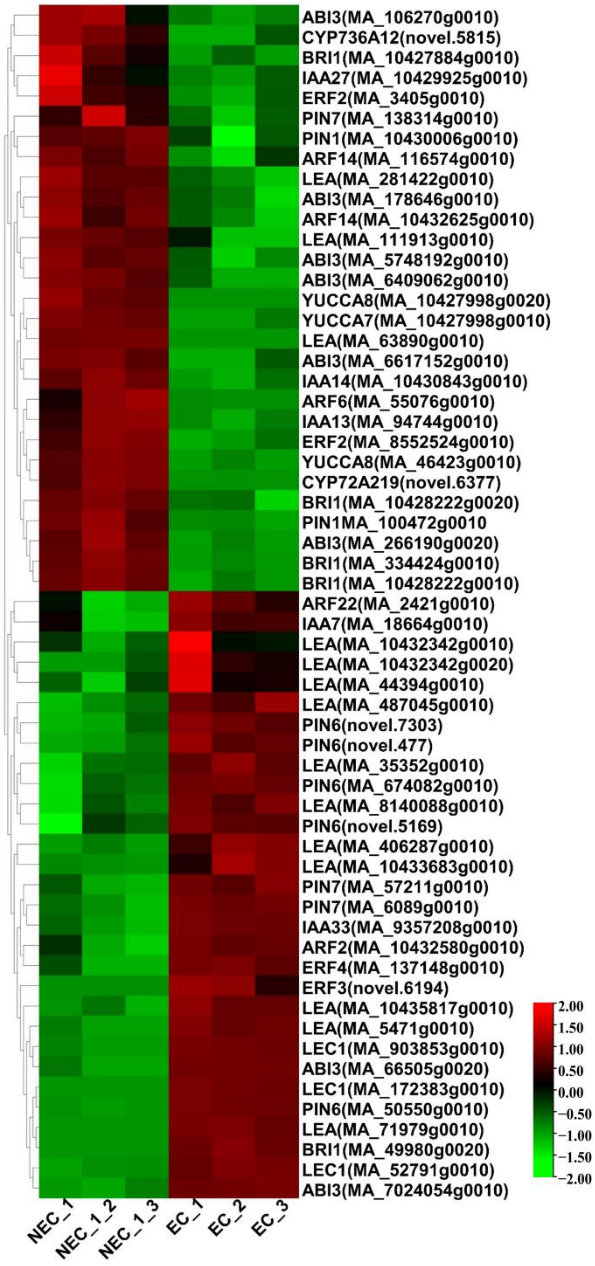

Figure S8. Heatmap of the DEGs related to TFs annotated to SE in EC vs NEC.
