# Supplementary material for: Transcriptome Analysis of Embryogenic and Non-Embryogenic Callus of Picea Mongolica
Source: Curr Issues Mol Biol. 2023 Jun 21;45(7):5232–47. doi: 10.3390/cimb45070332 (PMC10378709; doi:10.3390/cimb45070332)
Supplement: Supplementary file 1 [file cimb-45-00332-s001.zip › cimb-2448808-supplementary/Tables S1-S2.pdf]

Table S1. Medium composition table

**Component (concentration)**	**Induction Medium**	**Proliferation Medium**	**Differentiation Medium**
Sucrose (g/L)	20	20	30
Inositol (g/L)	0.2	0.2	0.2
Macroelement (ml/L)	80	40	40
Calcium salt (ml/L)	80	40	40
Microelement (ml/L)	10	10	10
Organic Element (ml/L)	10	10	10
ferric salt (ml/L)	10	10	10
Casein acid hydrolysate (g/L)	1.0	1.0	1.0
PEG4000 (g/L)	0	0	42.5
2，4-D (mg/L)	4.0	2.0	0
6-BA (mg/L)	2.0	2.0	0
KT (mg/L)	1.0	0.5	0
ABA (mg/L)	0	0	2.2
Phytagel plantcell (g/L)	1.25	1.25	4.5

Table S2. qRT-PCR primer information

Gene Name	F-primer	R-primer
EF1α	GCGTGCTGAAACTGGTGT	TTGGGTGATGCTTCTAGTGGAG
GST	TCTTAGGTGTGGCTCGGAGT	CTTCCATGCCTCCTCAATGT
GLP	TGAAGGCTCTTCCACTGGTT	ACGCTCAGAGCCAATGTCTT
LEC	GTGCCAAAAGGAACAAAGGA	GGGCATCTCCTCTGATTGAA
WOX9	AGCATTGGCCCAGTATGTTC	TTCCATCTGGGCTTTGTTTC
SERF	AGAAGAGGCAACGTGAGGAA	TCCACGGGATAAGAAACCAG
BBM	ATTCCCTGAATGCACTTTGC	TCTTTTGGCTGCTCAGGATT
GH3	GCCATTCAATGCCCTGTTAT	CAAACGTCCCAGGTTTCACT
AP2	ATCCCTCCTCCTCCTCACAT	CCTCCACAACTACACGCTCA
MYB	AATGGGCACAACTCCAGAAC	TGTTCTTCTTCCGTCCAAGG
